# A simple mathematical model utilizing topological invariants for automatic detection of tumor areas in digital tissue images

**DOI:** 10.1186/1746-1596-8-S1-S27

**Published:** 2013-09-30

**Authors:** Kazuaki Nakane, Yasunari Tsuchihashi, Nariaki Matsuura

**Affiliations:** 1 Osaka University, the graduate school of medicine, division of health science, Yamadaoka, Suita, Osaka, 565-0871, Japan; 2 Louis Pasteur center for medical research, Kyoto, department of clinical pathology research, Taniguchi Kakinouchi-Cho, Ukyo-Ku, Kyoto, 616-8012 , Japan

## Background

Pathological diagnosis starts usually from detection of morphological deviations of tissues and cells in question from their normal counterparts. Measurement and analysis of how they are deviated are the processes of actual pathological diagnosis and many of them can be achieved by computer using certain mathematical models. Computer assisted pathological diagnosis is now an issue of importance in the current situations of shortage of diagnostic pathologists in Japan. In the present study we propose a simple mathematical model to differentiate tumor tissues from their normal counterparts utilizing changes in *the Betti numbers* in tumorigenesis. We tested our method in several normal and tumor tissue images and preliminary results are reported.

## Materials and methods

### Mathematical tools

*The Betti numbers* are the numbers coined after Enrico Betti, an Italian mathematician of topology. *The Betti numbers* are one of the invariants in a homology. The invariant implies the quantity that is unchangeable by continuous transformation ([1]).

**Figure 1:**
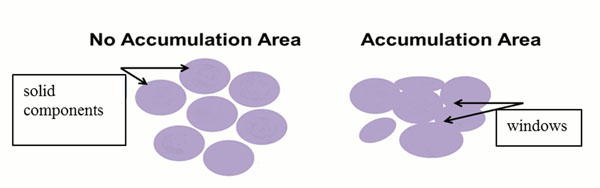
**The schematic illustration.** Left: the number of solid components *b0* =7 and the number of windows *b1*=0, then the ratio *b1/b0*=0/7=0, Right: the number of solid components *b0*=2 and the number of windows *b1*=4 and the ratio *b1/b0*=4/2=1.5.

We treat two dimensional pathological images, *the Betti numbers* are consisting of two numbers. One is *b0* (*the 0-dimensional Betti number*), which is the number of such isolated solid component as each cell or cell nucleus. The other is *b1* (*the 1-dimensional Betti number*), which is the number of windows in the fenestrated area. That area is created by incomplete fusion of neighboring isolated solid component. The schematic illustration is shown in Figure 1.

### Morphological changes during the course of tumorigenesis

Normal cells are usually controlled so as to grow and be reproduced only when a tissue composed of the cells requires new cells. That is, a new cell is generated when the old cell dies or is damaged. However, if the foregoing process of cell growth and cell division becomes out of order, the cells grow and divide, excessively. The excessive cells then produce a mass of tissues called “a tumor” or “a neoplasm”. The disordered cell growth causes a variety of morphological characteristics of tumor tissues.

### Mathematical representation of morphological change in tumorigenesis

Uncontrolled cell growth usually makes cell nuclei become larger. This causes a change in contact condition between components in a tissue. This change appears along with neoplastic changes and tumorigenesis. The following description deals with how to numerically express the changes.

As shown in Figure 1, when the number of contact points between individual solid components (cells or cell nuclei) increases, i.e., 7 solid components (*b0*) fuse each other through contact, 2 solid components with fenestration are created. They have 4 windows (*b1*). Namely, *b1* increase and the ratio *b1/b0* significantly change.

## Numerical procedure of our method

Hematoxylin and eosin stained mucosal biopsy sections of colonic tumors are used as test samples. First they are taken as whole slide images and then binarized setting a threshold value depending on each HE stained condition. We regard the binarized image as a sum of *a simplical complex*. By using a free calculation software (cf. [2]), we can calculate *the Betti numbers*. We compare the values, *b1* and *b1/b0*, of colonic tumor tissues with those of normal tissues.

**Figure 2:**
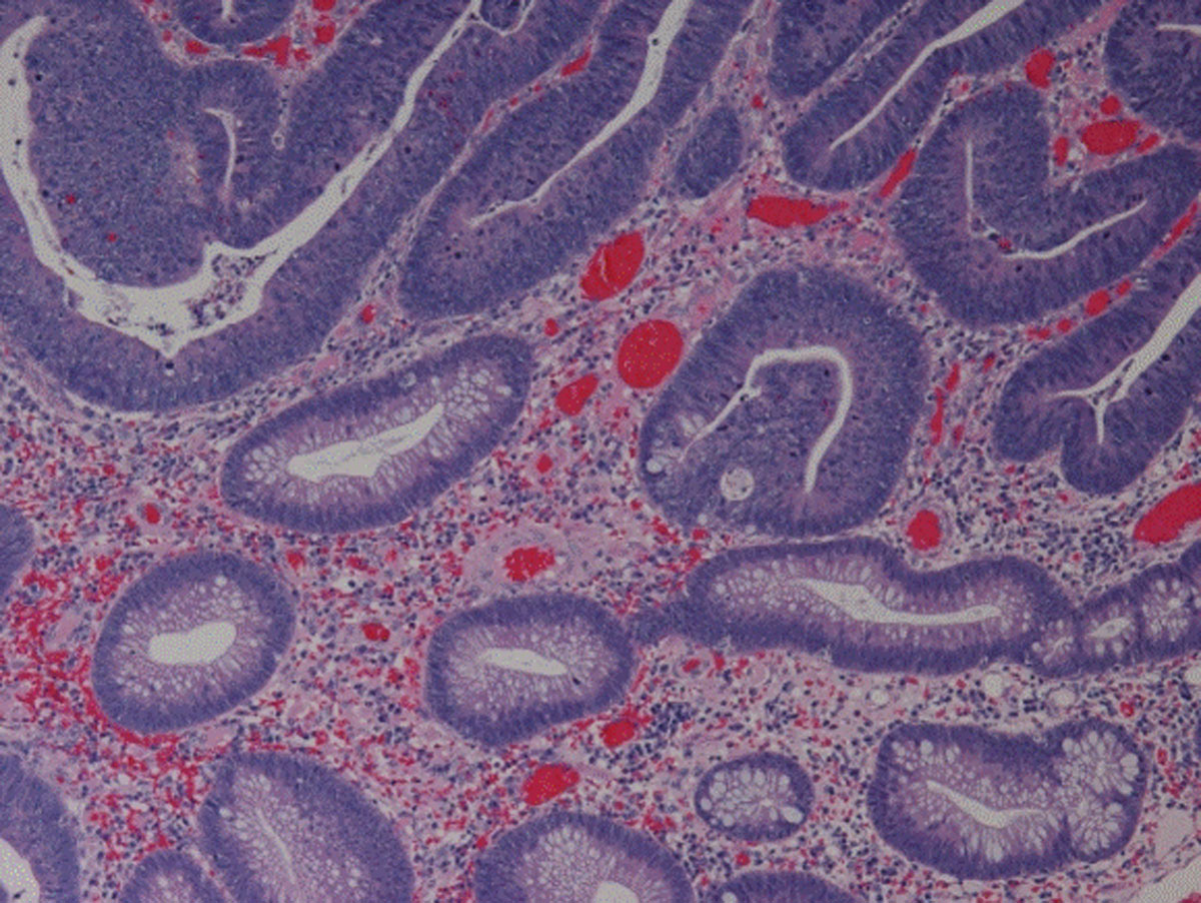
A digital image of tissue of colon.

In Figure 2, we show a digital image of tissue of colon and its binarized image. In the tumor area, we can see many windows.

## Results and discussion

*The Betti numbers* are obtained per unit binarized test image areas of normal and tumor tissues of colon. The values, *b1* and *b1/b0*, of representative each five unit areas of normal and tumor colonic tissues are listed in Table 1.

**Table 1:** *b1* and *b1/b0* of the normal and tumor tissue.

Normal tissue			Tumor tissue		
	*b1*	*b1/b0*		*b1*	*b1/b0*

a015	1300	0.496	a012	4045	2.16

a017	233	0.106	a034	3469	2.65

a045	903	0.204	a047	2377	5.23

a049	283	0.204	a067	3044	5.92

a050	109	0.0689	a070	3397	3.02

Ave.	565.6	0.251	Ave.	3266.4	3.79

In normal tissue, *b1* is less than 1400 (565.6 average), *b1/b0* is less than 1.4 (0.251 average). If tissue is malignant, *b1* is large (3266.4 average), *b1/b0* is increased (3.79 average).

Based on the results obtained above, the indices for a normal tissue and a tumor tissue can be assumed.

**Figure 3:**
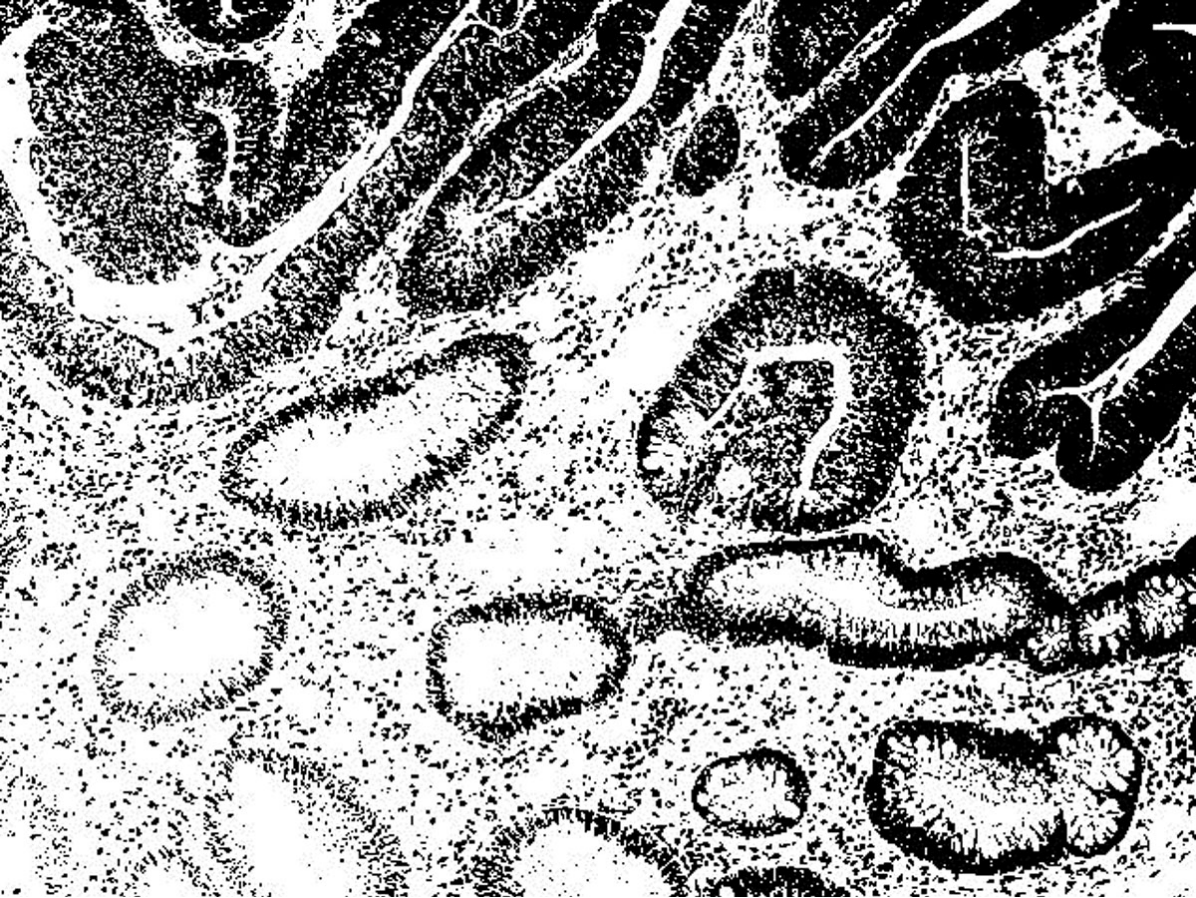
The binarized image of Figure 2.

Figure 3 is a result of this method. The image is divided into 7×7 unit areas and the marks are put on each unit area as Table 2.

**Figure 4:**
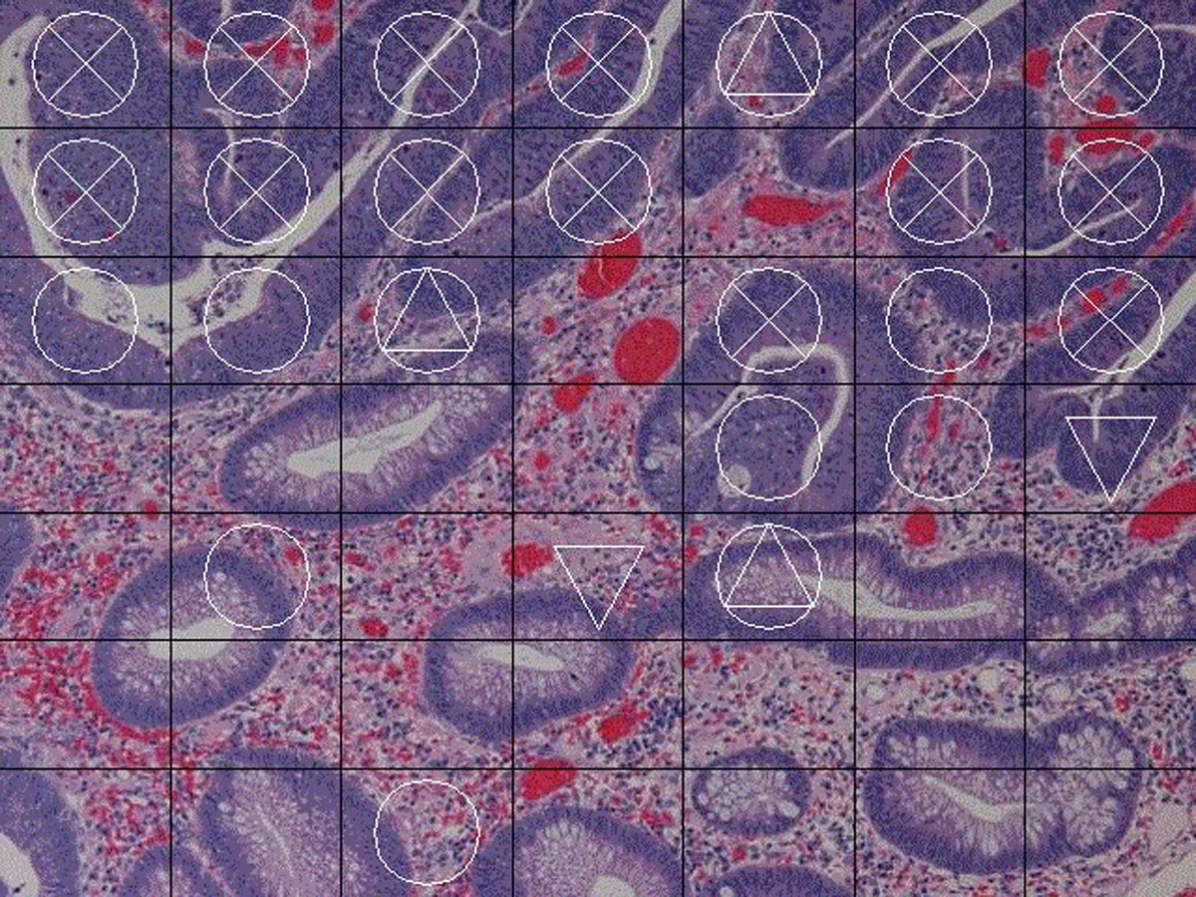
The image is divided into 7×7 and impose the marks as the result.

**Table 2:** Marks are put on each unit area as the value of index

*b1*	None (~ 1400)	▽ (1400 ~ 2000)	× (2000 ~ )
*b1/b0*	None(~ 1.4)	△ (1.4 ~ 1.7)	○ (1.7 ~ )

## Conclusion

*The Betti numbers* can express the degree of the connection of the figure. When the number of contact points between individual components increases, irrespective of their shape, *b1* and a ratio *b1/b0* significantly change. So we hypothesize that these numbers can be used as indexes to represent the cellular “accumulation” which is one of the characteristics of tumor tissue.

The numerical results indicate that the difference in our indices can differentiate tumor tissues from their normal counterparts.

## Competing interests

The authors declare that they have no competing interests.

## Authors’ contributions

KN carried out most of experiments, participated in the design of the study and drafted the manuscript. TY and MN participated in the design of the study and helped the manuscript. All authors have read and approved the final manuscript.
